# Molecular studies of exercise, skeletal muscle, and ageing

**DOI:** 10.12688/f1000research.8255.1

**Published:** 2016-06-02

**Authors:** James A. Timmons, Iain J. Gallagher

**Affiliations:** 1 Division of Genetics & Molecular Medicine, King's College London, London, UK; 2 School of Health Sciences, University of Stirling, Stirling, UK

**Keywords:** human muscle biology, ageing, excercise, skeletal muscle, human physiological capacity

## Abstract

The purpose of an F1000 review is to reflect on the bigger picture, exploring controversies and new concepts as well as providing opinion as to what is limiting progress in a particular field. We reviewed about 200 titles published in 2015 that included reference to ‘skeletal muscle, exercise, and ageing’ with the aim of identifying key articles that help progress our understanding or research capacity while identifying methodological issues which represent, in our opinion, major barriers to progress. Loss of neuromuscular function with chronological age impacts on both health and quality of life. We prioritised articles that studied human skeletal muscle within the context of age or exercise and identified new molecular observations that may explain how muscle responds to exercise or age. An important aspect of this short review is perspective: providing a view on the likely ‘size effect’ of a potential mechanism on physiological capacity or ageing.

## Introduction

Identification of effective strategies to substantially improve ‘health span’ in humans would revolutionise current approaches to health care. Rather than the current focus on the development of diverse therapies for treating *individual* age-correlated diseases (cancer, heart disease, and dementia), a strategy that positively impacted on the ageing process would in theory postpone disease. During this extended period of absence from illness, sustained functional capacities (for example, muscle strength and cognitive status) would be key components of ‘healthy ageing’. In turn, these two functional capacities (or approximate correlates) represent key clinical measures that are often used to define the ageing process. There is extensive evidence that exercise capacity (an integrated property of neuromuscular, metabolic and cardiovascular function) is under strong genetic as well as environmental influence. Indeed, key components of exercise capacity (for example, aerobic capacity) vary regardless of the observed range of exercise behaviours ^1^ in older adults.

In fact, the role of genotype influencing ‘intrinsic’ exercise capacity coupled with the impact of genetics on adaptation to exercise training ^2^ is not the only complication for studying human ageing. The impact of population stratification on the interpretation of cross-sectional analyses exploring the mechanisms for musculoskeletal ageing is typically under-appreciated. For example, over 30% of all cancer-related deaths occur before the age of 64 years ( www.cdph.ca.gov), meaning that this subpopulation of individuals can never be included in a cohort of subjects who were at least 70 years old. Notably, the molecular biology of tumour growth and muscle responses to exercise share many common effectors (for example, mechanistic target of rapamycin [mTOR] signalling and pro-angiogenic genes). Furthermore, the ever-popular research strategy ^3^ of comparing the health (or physiology) of sedentary older people (at least 70 years old) with life-long exercisers does not represent an easily interpretable experimental design (for genetic and behavioural reasons). Thus, studying the interaction among individual rates of biological ageing ^4^, exercise behaviour, and molecular factors, including genetics, is clearly non-trivial, and greater caution must be shown when claiming that exercise *per se* impacts on ageing or health in the elderly. In the present review, we selected about 50 articles from the more than 200 titles published during 2015 that refer to the topic of ‘skeletal muscle, ageing, and exercise’ with the aim of highlighting new understanding or research methods in addition to illustrating technical issues which we feel represent barriers to progress. Studies included further, robust illustration of the highly variable metabolic adaptation noted in response to supervised exercise training ^5^.

## Recent human studies that address controversies in exercise, ageing, and muscle frailty

The Kraus laboratory ^5^ demonstrated that improvements in insulin action occurred in fewer than half of overweight subjects undertaking high-volume aerobic or resistance training. Earlier observations found that only the highest training load (something equated to the integral of time/volume) of combined aerobic and resistance training provided a measureable reduction in the group average for indices of glycaemic control ^6^. Interestingly, a correlative analysis this year found that vigorous exercise was most closely associated with modest reductions in all-cause mortality ^7^, and notably combined resistance and aerobic training will result in the exercise sessions including more ‘vigorous’ muscle contractions than those encountered during walking or jogging. Nevertheless, older people in better health (for genetic or other reasons) will be *able* and potentially may be more *willing* to undertake vigorous daily physical activity, more so than their unhealthy peers, and this can explain the epidemiological correlation between exercise and health in the elderly. To progress our understanding of the true relationship between ‘exercise’ and health, we need to establish causal mechanisms through randomised clinical trials coupled with global molecular analyses. Such studies will also help evolve a more personalised approach to public health.

Use of the term sarcopenia, which refers to ‘age-associated loss in muscle tissue’, is relatively new ^8, 9^ and there has been limited progress in identifying the mechanisms driving sarcopenia in humans. Although both nutrition and exercise habits are postulated to contribute to the prevalence of sarcopenia ^10^, recent studies state that the overall prevalence remains rather low (approximately 5% in patients who are at least 70 years old), but this may reflect the criteria used and the prevalence may be three times greater (but still far from universal). The prevalence of sarcopenia also increases with diseases that impact on mobility ^11^. With respect to inherent defects within the muscle contributing to sarcopenia, the van Loon laboratory found that post-prandial muscle protein synthesis was reduced by approximately 16% in healthy older men versus young men ^12^, independently confirming the anabolic resistance concept first identified by Smith and Atherton ^13^. It is interesting to consider whether the process of sarcopenia is substantially (quantitatively) influenced by insulin resistance. In 2010, it was reported that among 810 Korean subjects, the prevalence of one definition of sarcopenia was approximately 16% in subjects with diabetes versus approximately 7% in the control subjects by 60 years of age ^14^. Others have reported that lean mass is approximately 10% lower in older subjects (about 73 years old) with diabetes and that the rate of loss of computed tomography (CT)-determined thigh cross-sectional area is greater in women, but interestingly not in men, with diabetes ^15^. Whether this is reflective of the molecular features of skeletal muscle in diabetes ^16^ or a consequence of physical activity is less clear (given the lack of direct measures of exercise); the molecular features of sarcopenia, insulin-resistance, and gender need to be compared in detail.

Recently, a meta-analysis by Markofski *et al*. noted that young and old skeletal muscle does not differ in *basal* (fasting) protein synthesis rates but that both total and phosphorylated mTOR (a protein complex influencing protein synthesis and breakdown) were elevated in muscle from fasted elderly individuals ^17^. This may reflect a compensatory response, reflecting increased *relative* muscle loading in daily life (due to reduced muscle strength or conditioning) or may reflect dysregulation of the mTOR complex, which influences muscle mass in a complex and non-linear manner ^18^. Kirby *et al.* noted that ‘elderly’ mice subject to synergist ablation had lower transcription of ribosomal genes (downstream of mTOR signalling) compared with young mice and that ribosomal gene transcription returned to baseline faster in the old mice ^19^. In model organisms, the mTOR inhibitor rapamycin can extend lifespan. Whilst acute rapamycin administration reduces translation, data this year indicated that chronic administration does not ^20^. More work on the mechanisms underlying rapamycin’s effect on lifespan (mTOR mediated and mTOR independent) as well as the relevance of changes in translation is required.

Therefore, it is still plausible that older people with less sarcopenia start off with greater mobility (or less decline) promoting retention of muscle mass, rather than the development of 'sarcopenia' being the initiating factor. Adding support to this idea, Venturelli *et al.* ^21^ found that the properties of isolated muscle fibres remained intact in frail older people, while Hart *et al.* ^22^ found that when physical activity levels were matched (and oxygen delivery capacity was similar), no impact of ageing on plantar flexor oxidative metabolism was noted, suggesting no ‘inherent’ loss of mitochondrial function with age in human muscle. Given the modest prevalence (5 to 15% of those over 70 years old depending on the criteria selected), the contribution of sarcopenia to musculoskeletal frailty (impaired function) in old age remains unquantified. Further, it seems to us that the role for insulin resistance (or obesity) ‘causing’ sarcopenia or frailty has potentially been overstated and only quantitatively modest associations have genuinely been observed. For example, Kim *et al*. ^23^ found that CT inter-quartile values for thigh cross-sectional area were 1.7 to 2.1 in 50 year olds with normal weight and homeostatic model assessment insulin resistance (HOMA-IR) but were 1.5 to 2.0 in age-matched obese subjects with insulin resistance – hardly a striking difference. Indeed, after studying 482 adults, Loenneke and Loprinzi found that lean body mass was maintained in the face of insulin resistance and inflammation ^24^. Overall, this topic is challenging to address in cross-sectional analyses and using relatively blunt instruments (such as dual-energy X-ray absorptiometry [DXA] or indeed physical activity questionnaires).

One widely accepted strategy for tackling muscle wasting and weakness is supervised resistance exercise training. Nevertheless, it is still debated whether older people (at least 70 years old) respond in a manner similar to that of younger adults ^25^. Population stratification (as a consequence of ‘premature’ deaths, e.g. cancer) complicates the evaluation of the efficacy of resistance training with respect to human age. This is because up to 25% of healthy adults fail to demonstrate any hypertrophy response to long-term resistance training ^18, 26^. Interestingly, a study this year described a lack of *hypertrophy* response to 6 months of resistance training in a group of otherwise healthy and physically active elderly women (about 68 years old), while on *average* muscle strength increased by 20% ^27^. The profile of non-responders for gains in function was not reported. The development of diagnostics that could ascertain the proportion of non- or low-responders to resistance training in sarcopenic elderly populations would help clarify the association between molecular potential to ‘grow’ muscle and ability to maintain function with age. Too many human physiology studies undoubtedly report spurious observations owing to small sample sizes and high inter-subject variation, while the use of cross-over designs requires knowledge of appropriate ‘wash-out’ periods. A valid diagnostic could be applied at any age to create a profile of an individual, and this could be used to create better-matched ‘case versus control’ cohorts, each with equal potential for ‘trainability’ or with similar ‘intrinsic’ exercise capacities, when evaluating nutrition or lifestyle interventions.

However, it must be pointed out that not all researchers agree on the scale of heterogeneous outcomes obtained from exercise training. The idea that weeks (12 to 24) of supervised resistance training could yield ‘non-responders’ was challenged this year ^28^, using retrospective analysis of about 200 older men and women. The claim that there were no ‘non-responders’ to resistance exercise training was based on the observation that *at least* one of six (inter-related) laboratory parameters changed in every subject (multiple strength measures, muscle fibre diameter, and lean mass variables). For example, isolated quadriceps muscle function was generally improved in all subjects. However, with an everyday physical test of function (chair-rise time), where accommodation (task learning) is less of an issue, there were non-responders. The study did not formally consider test-retest variation or accommodation or adjust for multiple *t* tests. Furthermore, although gains in integrated muscle function—reflecting improvements in multiple physiological factors (for example, coordination, muscle mass, and neuromuscular function)—may be more common to most *healthy* subjects with exercise training, whether this is true for older subjects with pre-existing skeletal problems or disease (for example, diabetes) is less clear ^25^. This returns us to the issue of population stratification, a key challenge for the field to address, ideally using larger-scale longitudinal studies and improved diagnostics of an individual’s potential to respond to standardised physical training.

## Connecting the dots: acute responses that convey chronic changes in muscle function

There is an expectation that the molecular and metabolic events that occur during and in the minutes after a *single* bout of exercise will, over the period of weeks to months, ‘accumulate’ to cause a change in physiological capacity (for example, maximal oxygen consumption [VO _2_ max] and improved insulin-mediated glucose clearance). Notably, this ‘accumulative’ concept is somewhat contradicted by data from human studies ^29^. Rather than linear accumulating adaptive changes, change may occur through seemingly unrelated secondary molecular responses distinct from acute molecular responses and, critically, be greatly influenced by background genetic and epigenetic factors. There is in fact some pre-existing evidence that muscle molecular responses to acute-on-chronic exercise are rather stochastic ^29^, and this remains one of the most active and challenging topics to study in exercise physiology.

Critically, work in this area has, to date, failed to take advantage of the inherent heterogeneous ability for out-bred mammals to chronically adapt in the face of regular bouts of exercise ^30, 31^. In short, acute studies should be carried out in subjects that have been characterised (either by diagnosis or following training + wash-out) for their ability to chronically adapt to the same exercise paradigm. If you accept that the much-ignored issue of population stratification in the older population can result in a different outcome than seen in younger adults (i.e. 75-year-old people are not a random sample of all younger adults), then it is feasible that individuals presenting with musculoskeletal frailty in older age are also those who are less able to mount a muscle hypertrophy response ^18^ or have a more blunted response to nutrition throughout life ^13^ or both. Thus, we will need larger, more sophisticated studies, as well as studies of longer duration, to properly investigate what aspects of musculoskeletal ageing are related to heterogeneous responses to exercise training or nutrition ^1^.

This year saw the continued search for key molecular ‘targets’ that may govern skeletal muscle size or function ^32^. Most new studies in 2015 focused on *consistent* molecular responses (group mean differences identified by a paired *t* test) or used inbred murine models, where variability is understandably much more limited ^33^. It may be more fruitful to stratify acute response against measures of physiological adaptation ^34^. In 2015, two contrasting approaches considered 5′-AMP-activated protein kinase (AMPK) as a candidate transducer for acute-to-chronic adaption. The Wojtaszewski group found that functional gains (endurance capacity) with 4 weeks of training in mice were the *same* with or without muscle AMPKα1/2 ^33^ but that acute responses in vascular endothelial growth factor ( *VEGF)* mRNA, a gene involved in vascular adaptation ^35^, was *apparently* reliant on AMPK. This latter interpretation is probably unreliable, as the AMPK knockout (KO) mouse did 40% less work during the acute bout and, surprisingly, despite also being approximately 50% less physically active, it retained normal insulin and glucose control ^36^. It is plausible that 50% of normal physical activity in ‘cold mice’ ^37^ is sufficient to maintain good metabolic health (owing to the very high metabolic turn-over), and careful energy balance studies are needed to establish whether the room-temperature-housed mouse model can ever illuminate mechanisms relevant to human metabolic health.

Genetic and most probably epigenetic factors can influence exercise behaviour and capacity ^38, 39^ (for example, altered levels of voluntary wheel running [mice] or aerobic capacity). However, the appearance of specific phenotypes in genetically manipulated mice may or may not reflect the intended genetic manipulation. Indeed, controlling variables beyond the targeted gene when generating transgenic murine models was always deemed complex ^40^ and nearly impossible to achieve with complete certainty, while newer gene editing technology (that is, CRISPR/Cas9) still has off-target concerns ^41^ and detection of individual ‘secondary’ molecular alterations is challenging to spot, as studies are not powered to detect each slight off-target event (but additively they can appear as altered physiology). However, the absence of *any* effect on chronic adaptation does suggest that AMPK, like PGC1a before it ^42^, is not an essential determinant of physiological responses to exercise. Of course, if hundreds of clinical studies characterise AMPK or PGC1a, then chance associations will still be found (and will be more likely to be published). Nevertheless, we would encourage investigators to move beyond characterising these ‘usual suspects’. In this context, more global (‘unbiased’) research tools provide an advantage, particularly when analysed in a responsible manner ^43^. Such approaches are more likely to uncover more subtle molecular signatures as statistical models (which accumulate small interactions) can be generated, a situation that arguably reflects physiological control.

A recent human study profiling a substantial part of the phosphoproteome identified AMPK-regulated kinases as being the *consistent* feature of the acute intense cycle exercise in younger males ^34^. This study quantified more than 4300 proteins in human muscle (note that there are about 40,000 RNA molecules detectable in human muscle), identifying many canonical signalling proteins (for example, MAPK, PKA, S6 kinase, and Ca ^2+^ response kinases) and about 600 phosphopeptides that were modified (with *mean* response statistically consistent). Encouragingly, from our perspective, they found that about 50% (that is, 50% of the shared variance) of the phosphopeptide response to acute exercise was *inconsistent* across subjects, creating the possibility that studying the highly variable phosphopeptide responses to muscle contraction can help identify factors which contribute to variation in physiological adaptation, if such factors are in operation during acute contraction.

The caveat to phosphopeptide measurement technology is that at this stage, the overall technical reproducibility of the methodology has not been established. Given that the same researchers have demonstrated that murine muscle AMPK is not essential for physiological adaptation to exercise training ^33^, or fasting glucose and insulin homeostasis for that matter, then the human phosphopeptide responses which are *unrelated* to AMPK could transpire to be the most interesting to characterise in future human studies. A more detailed discussion of considerations when applying proteomics to the study of muscle and exercise can be found in Padrão *et al.* ^44^, and this review indicates that the technology is very much a ‘work in progress’. In general, alternative statistical analysis strategies applied to existing data would be beneficial. However, an important challenge is placing the molecular responses within a familiar biological context, as a valid pathway or ontology enrichment (although used) is not possible with ‘proteome’ data because of a lack of knowledge of the detectable background (or ‘universe’) of molecules. Without such information, the regulated list is merely a subsample of the tissue being studied and invariably yields significant enrichment scores versus the ontology database ^43^. Stratification of detected molecules against physiological phenotype seems to us the most productive strategy for human studies in the shorter term.

## Non-coding RNA that has potential to impact on the biology of exercise, skeletal muscle, and ageing

Among the most exciting molecular entities contributing to our understanding of the complexity of the genome and physiological regulation are long non-coding RNAs (lncRNAs). Like microRNAs (miRNAs), these transcribed RNAs do not, usually, code for protein. Rather, the transcribed product represents the active molecular effector, regulating the processing of other RNA molecules ^45^ and hence influencing protein expression. lncRNAs are not new to skeletal muscle and exercise; it has been almost a decade since we identified that the *PINK1* antisense RNA was regulated by endurance training in humans, altering splicing of *PINK1*, a metabolic gene linked to Parkinson’s disease ^45^. What has changed is the verified scale of lncRNA expression ( www.noncode.org), and this includes human muscle tissue (for example, we routinely detect about 9000 lncRNAs by using Affymetrix HTA 2.0 arrays; Affymetrix, Santa Clara, CA, USA). The Glass laboratory recently described *LncMyoD*, a gene able to control myoblast proliferation and hence potentially influence muscle regeneration from damage ^46^. They identified more than 1000 intergenic lncRNAs in the murine C2C12 cell line by using RNA sequencing data, many regulated during myotube formation (it would be interesting to know whether the diversity of expressed lncRNAs is greater in primary muscle cells to better match muscle *in vivo*). Knockdown of *LncMyoD* hampered myogenesis, reducing the expression of genes expressed in mature muscle cells ^46^, indicating a role for *LncMyoD* in muscle development and hence potentially in recovery from injury.

One benefit of research reliant on the detection of RNA molecules is that with a little care ^47^, you can be sure of the identity of the molecule you are quantifying (a situation far removed from antibody-based technologies; see below). However, in the case of lncRNAs, the situation is not nearly as simple as one might hope. The Olson laboratory this year described a lncRNA related to murine exercise performance, but the ‘non-coding’ RNA in this case contained a conserved open reading frame that coded for a micropeptide that played a role in regulating calcium handling in murine ‘fast skeletal muscle’ ^48^. So, while one can be sure of the identity of the RNA molecule being measured, emerging evidence this year ^49^ indicates that a lot of the less-well-characterised ‘non-coding’ transcriptome may also contain uncharacterised coding regions, which in turn yield peptides that impact on physiological parameters ^46, 48^.

Discoveries in the field of miRNA biology continue and more families of these short (about 22 nucleotides) molecules have been associated with muscle age or found to be regulated by exercise or both ^50^. In humans, *in vivo* miRNA abundance does not strongly co-associate with target mRNA levels but rather protein abundance, which, in combination with transcription, regulates muscle phenotype ^16, 51^. In our experience, major challenges remain for the miRNA field, as the various technology platforms do not consistently measure each miRNA with the same sensitivity or specificity, and changes in abundance can be rather modest (supporting our model that miRNAs function in a combinatorial manner *in vivo* in adult human muscle and not individually). Thus, whilst *short* non-coding RNA molecules still represent a challenge for all high-throughput technologies and inconsistencies between the various manufacturers’ technologies must be expected (as well as lack of conservation of miRNAs between mouse and human), lncRNA molecules are easy to reproducibly measure and represent an exciting new set of molecules regulating muscle biology.

## Protein detection technologies as a barrier to research progress

Protein is considered by most physiologists to be the main effector of function in the cell, yet, unlike RNA, it is very challenging to quantify or specifically measure. In muscle biology, a particular importance is placed on quantification of protein abundance or enzyme activity reflecting the contractile and metabolic functions of this tissue and historical approaches to studying muscle physiology. This has led to a popular—and, we would argue, mistaken—focus on individual proteins (for example, AMPK) claimed to regulate complex physiological phenotypes and ageing ^52^. It is a simple fact that there are fundamental problems associated with how protein abundance or activity is quantified, and this has greatly impacted on the field of skeletal muscle biology and ageing. Such problems include ambiguity over the veracity of the reagents being used to quantify key protein factors associated with exercise or muscle ageing ^53^. For example, GDF11, a circulating growth and differentiation factor, was claimed to decline with age, ‘controlling’ muscle and vascular ageing ^54^, but was in reality misidentified ^55^. Pharmacologically, it appears that GDF11 can inhibit the potential contribution of skeletal muscle satellite cells to maintenance of adult human skeletal muscle ^56^ and so is plausibly related to human muscle ageing. In this case, both the GDF11 SOMAmer assay (SomaLogic, Boulder, CO, USA) and the GDF11 Abcam Western blot antibody (Abcam, Cambridge, UK) cross-reacted with myostatin ^55^, altering the described age-related changes in GDF11 in mice (while no significant changes were noted in rats or humans with age ^56^).

So how could such problems be avoided, and why is it a major barrier to progress? In the case of GDF11, experiments using tissue from GDF11 KO mice demonstrated that the ‘protein detection’ technologies were not uniquely detecting GDF11. This simple ‘litmus test’ for assay specificity (arguably the most useful thing one can do with a KO mouse) is not an obligatory prerequisite for publication, but this would be a useful development. Manufacturers could be obliged to provide the negative control tissues and cells to investigators. There is no doubt that lack of specificity of detection technologies yielding positive publications can also drive needless research efforts on the back of mistaken observations. For example, although the technical flaws around various GDF11 detection technologies have been in the public domain since July 2015, and there has been an ongoing and visible debate over the validity of *any* of the available regents ^57^, a raft of correlative studies using GDF11 antibodies have been subsequently published without any reference to the controversies ^58^. The scale of the problem is substantial; irrespectively of the publication of *unambiguous* evidence proving that the original lab reagents were faulty. For example, more than 250 articles reporting ‘measures’ of irisin were published between 2014 and 2016 despite multiple articles demonstrating the lack of suitability of available protein detection reagents ^59– 62^. Indeed, there is a substantial challenge in ‘pausing’ such activity once the proverbial ‘horse has bolted’. Therefore, failure to remedy high-profile discoveries ^59^ based on flawed protein detection technologies ^62^ is, in our opinion, a major barrier to research progress because follow-on research, motivated by mistaken observations or worse, is a considerable consumer of ‘resource’ (that is, researcher time and reagents) that could be better utilised to advance the field. If retrospective analysis reveals major flaws in published ‘impactful’ research, then editors could act more quickly to flag the problems with the original work, and in more clear-cut cases a ‘no-blame’ retraction should be quickly enforced. Indeed, if strong belief in the original research hypothesis remains, this should provide exceptional motivation to fully *replicate* the original study using validated reagents. This leaves the finite resources (people and funding) available to study human skeletal muscle and ageing better focused on more legitimate hypotheses or observations. Research into ageing, exercise, and muscle will by its nature be complex and involve multiple molecular measurement technologies, but further effort to improve the specificity of the available tools to quantify protein is, in our opinion, a pressing challenge that the field needs to address.

## Conclusions

The textbook principles that govern skeletal muscle physiology have until recently been developed on studying small homogenous unrepresentative healthy males ^63^, coupled with an over-reliance on acute exercise studies to study human skeletal muscle phenotype. Although it may be understandable (for example, financially), the lack of longitudinal studies with detailed phenotyping has also meant that our understanding of the impact of ‘ageing’ on human skeletal muscle is based on cross-sectional case-control studies. One general conclusion is that sooner rather than later there must be a shift away from funding small studies to funding larger longitudinal intervention studies capable of delivering more definitive answers. Finally, one might also argue that the greatest scrutiny should always be placed on any discovery that claims that a ‘single’ protein or gene can *quantitatively* explain ageing or exercise performance; extraordinary claims should always require multiple independent sources of evidence.
